# A Rare Case of Low-Grade Endometrial Stromal Sarcoma Invading an Old Leiomyoma

**DOI:** 10.7759/cureus.73329

**Published:** 2024-11-09

**Authors:** Kayo Inoue, Mizuho Kuroshima, Yuka Murata, Hiroki Morita

**Affiliations:** 1 Obstetrics and Gynecology, Konan Medical Center, Kobe, JPN; 2 Obstetrics and Gynecology, Kobe Children's Hospital, Kobe, JPN

**Keywords:** clinicopathological features, leiomyoma, low-grade endometrial stromal sarcoma, magnetic resonance imaging, positron emission tomography

## Abstract

We present the first case of low-grade endometrial stromal sarcoma (ESS) invading a leiomyoma, which was difficult to diagnose preoperatively. A 49-year-old multiparous woman was referred to our institution due to the enlargement of an old leiomyoma after menopause. Transvaginal ultrasonography revealed a 40-mm lesion in the myometrium of the uterine body with calcification and edema. Color Doppler imaging showed blood flow along the margins of the mass. Pelvic contrast-enhanced magnetic resonance imaging (MRI) revealed a 39-mm mass in the uterine body, predominantly having a low intensity on T2-weighted images, suggesting leiomyoma containing a cystic lesion with a small solid component. This solid component appeared to have a high intensity on T2-weighted images, high intensity on diffusion-weighted images, low value on apparent diffusion coefficient (ADC) map images, and contrast effect. ^18^F-fluoro-deoxyglucose positron emission tomography (FDG-PET)-computed tomography (CT) showed a non-significant FDG uptake in the cyst’s solid component. Based on a preoperative diagnosis of cystic or myxoid degeneration of leiomyoma, laparoscopic total hysterectomy and bilateral salpingo-oophorectomy were performed. The uterus was retrieved vaginally without morcellation.

Macroscopic examination of the hysterectomy specimens revealed a 55-mm white solid tumor with scattered yellow nodules. Histopathological analysis identified spindle-shaped smooth muscle cells with non-atypical nuclei, confirming a leiomyoma. However, the tumor’s nodules contained slightly atypical cells with round nuclei, resembling endometrial stromal cells interspersed with small blood vessels. Immunohistochemical staining showed the nodules were negative for alpha-smooth muscle actin and positive for CD-10, estrogen receptor, and progesterone receptor. These nodules invaded the leiomyoma along vascular vessels. The final diagnosis was leiomyoma coexisting with low-grade ESS, classified as International Federation of Gynecology and Obstetrics (FIGO) stage IB. The patient received no further treatment and remains disease-free after 45 months.

## Introduction

Low-grade endometrial stromal sarcoma (LGESS) is a rare uterine sarcoma that accounts for only 0.2% of all malignant uterine tumors [1]. LGESS is a tumor of endometrial stromal cells that usually invades the myometrium and may permeate it [2]. There are no prior reports of LGESS invading leiomyoma.

Differential diagnoses of myometrial cysts should include cystic degeneration of a leiomyoma [3] and rarely LGESS [4,5]. However, preoperative differentiation between LGESS and leiomyoma is difficult due to the unreliability of imaging studies [6].

Previously, we reported positron emission tomography (PET) features and tumor biology of recurrent or metastatic LGESS: low 18F-fluoro-deoxyglucose (FDG) uptake, immunohistochemical positivity for hormone receptors, and low Ki-67 index may be associated with favorable prognosis [7]. There have been no such reports in primary LGESS, making this the first report of PET features and immunohistochemical studies in a primary setting of LGESS.

Herein, we present the first case of LGESS invading a leiomyoma, which was difficult to diagnose preoperatively.

## Case presentation

A 49-year-old multiparous woman with a body mass index of 27.4 kg/m2 had been followed up by annual transvaginal ultrasonography for an asymptomatic myoma since her late thirties. At the age of 43, the lesion had a maximum diameter of 47 mm. The patient reached menopause at the age of 45 years, and it had shrunk to 17 mm at the age of 48 years, but it subsequently increased to 39 mm at the age of 49 years. The patient was referred to our institution.

**Figure 1:**
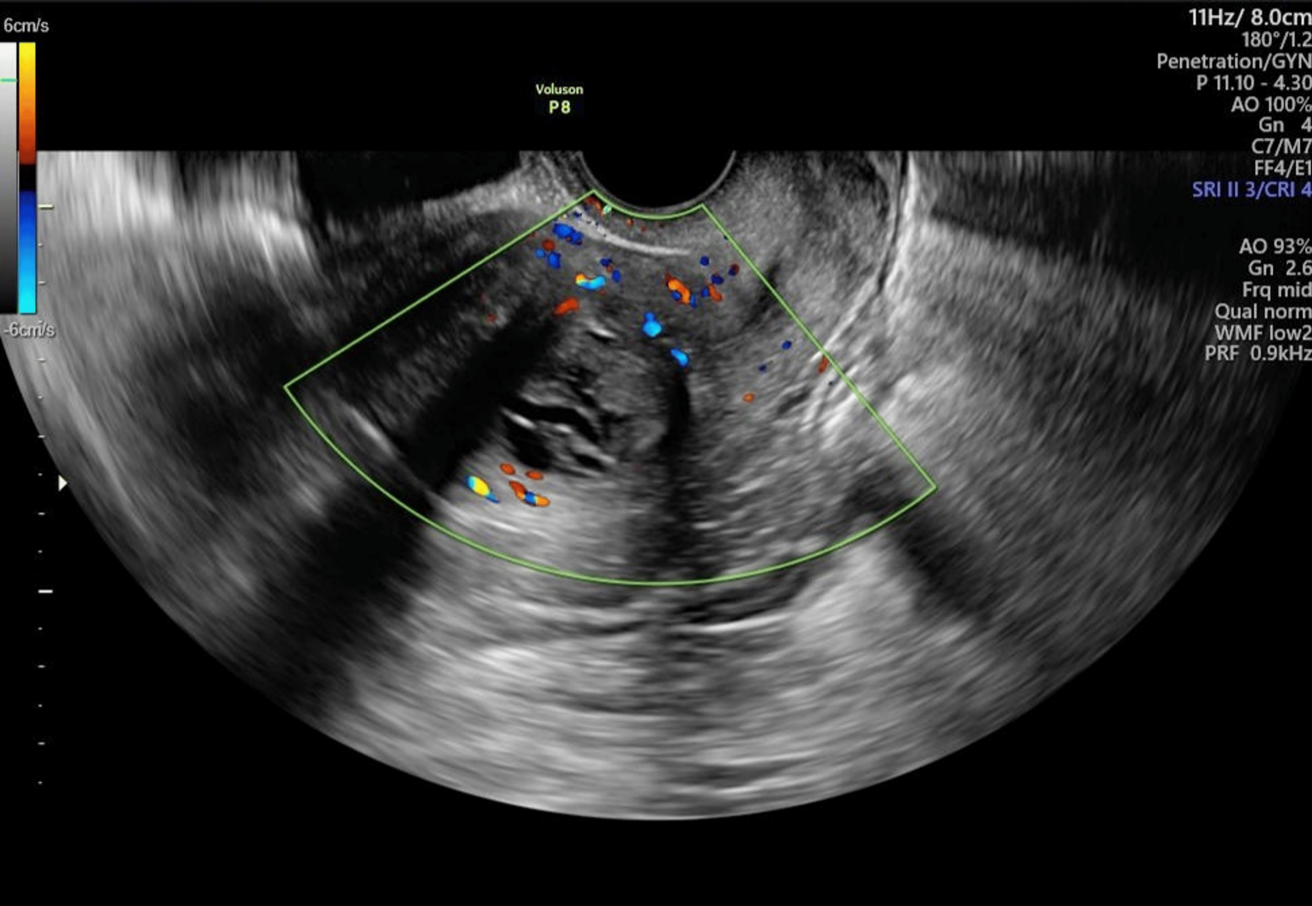
Transvaginal ultrasonography The image shows a 40-mm lesion in the myometrium of the uterine body with calcification and edema. Color Doppler imaging showed blood flow along the margins of the mass.

The patient had no symptoms at the time of her visit to our hospital. Transvaginal ultrasonography revealed a 40-mm lesion in the myometrium of the uterine body with calcification and edema. Color Doppler imaging showed blood flow along the margins of the mass (Figure 1). Cytological examinations of the cervix and the endometrium were negative.

**Figure 2:**
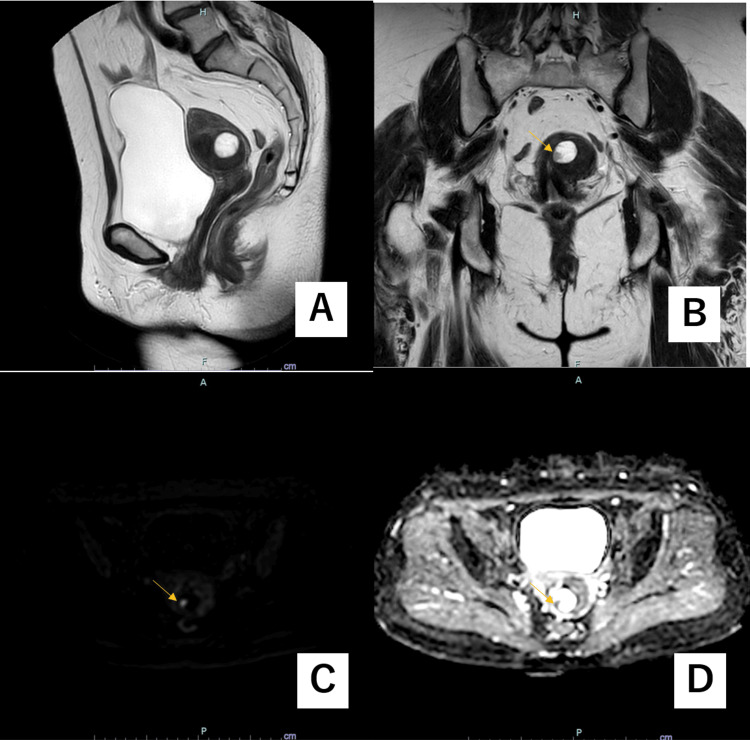
Pelvic contrast-enhanced magnetic resonance imaging The image shows a 39-mm mass in the uterine body, predominantly having a low intensity on T2-weighted images, suggesting leiomyoma containing a cystic lesion with a small solid component (indicated by the arrow).  This solid component appeared to be high intensity on T2-weighted images (A, sagittal, and B, coronal), high intensity on diffusion-weighted images (C, transaxial), and low value on ADC map images (D, transaxial). ADC, apparent diffusion coefficient

**Figure 3:**
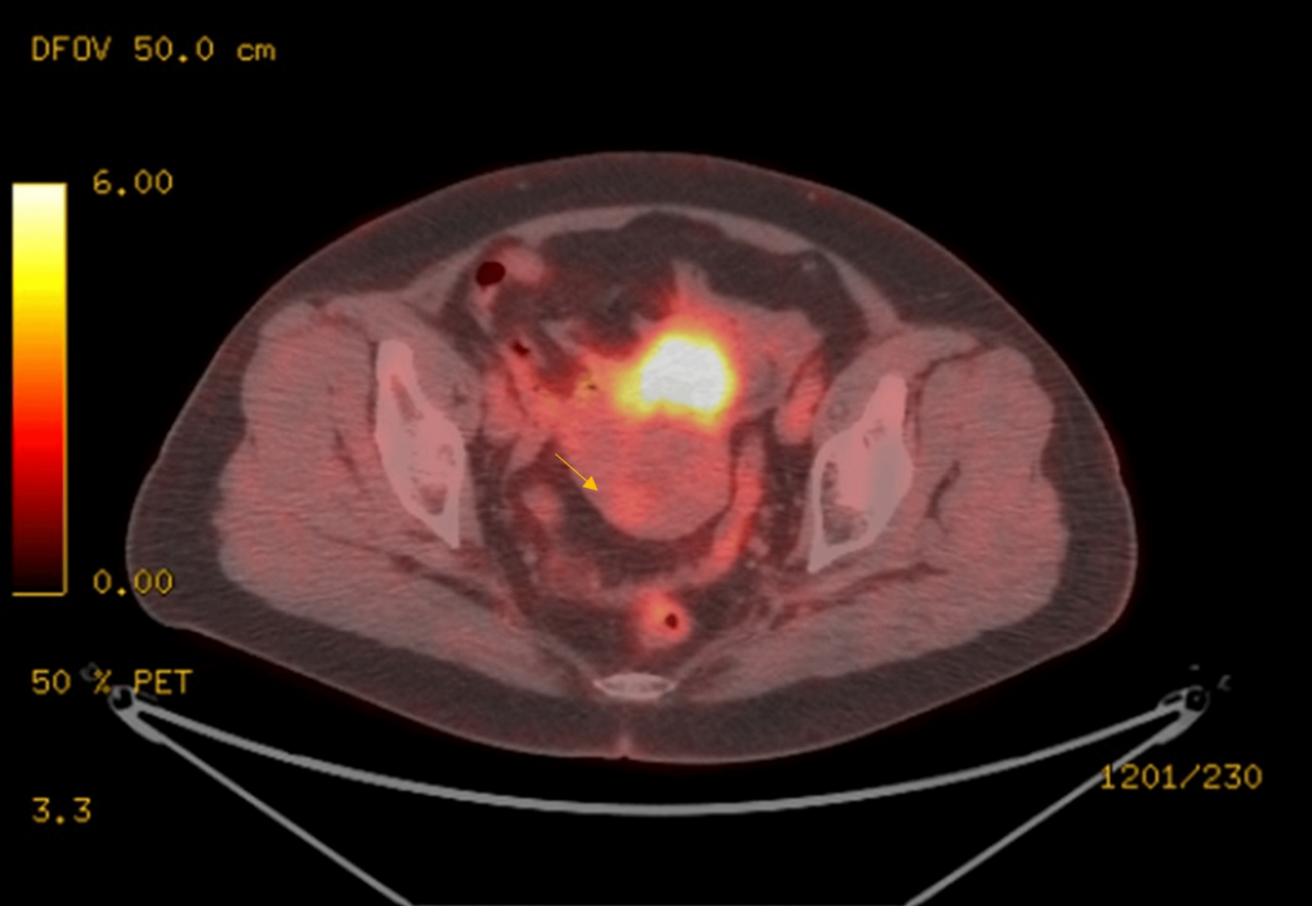
FDG-PET/CT A non-significant FDG uptake was observed in the cyst’s solid component (indicated by the arrow). FDG-PET, 18F-fluoro-deoxyglucose positron emission tomography; CT, computed tomography

Pelvic contrast-enhanced magnetic resonance imaging (MRI) revealed a 39-mm mass in the uterine body, predominantly having a low intensity on T2-weighted images, suggesting leiomyoma containing a cystic lesion with a small solid component. This solid component appeared to have a high intensity on T2-weighted images, high intensity on diffusion-weighted images, low value on apparent diffusion coefficient (ADC) map images, and contrast effect (Figure 2). To differentiate between degenerative myoma and uterine sarcoma, FDG-PET/computed tomography (CT) was performed. A non-significant FDG uptake was observed in the cyst’s solid component (Figure 3). Based on a preoperative diagnosis of cystic or myxoid degeneration of leiomyoma, laparoscopic total hysterectomy and bilateral salpingo-oophorectomy were performed. The uterus was retrieved vaginally without morcellation.

**Figure 4:**
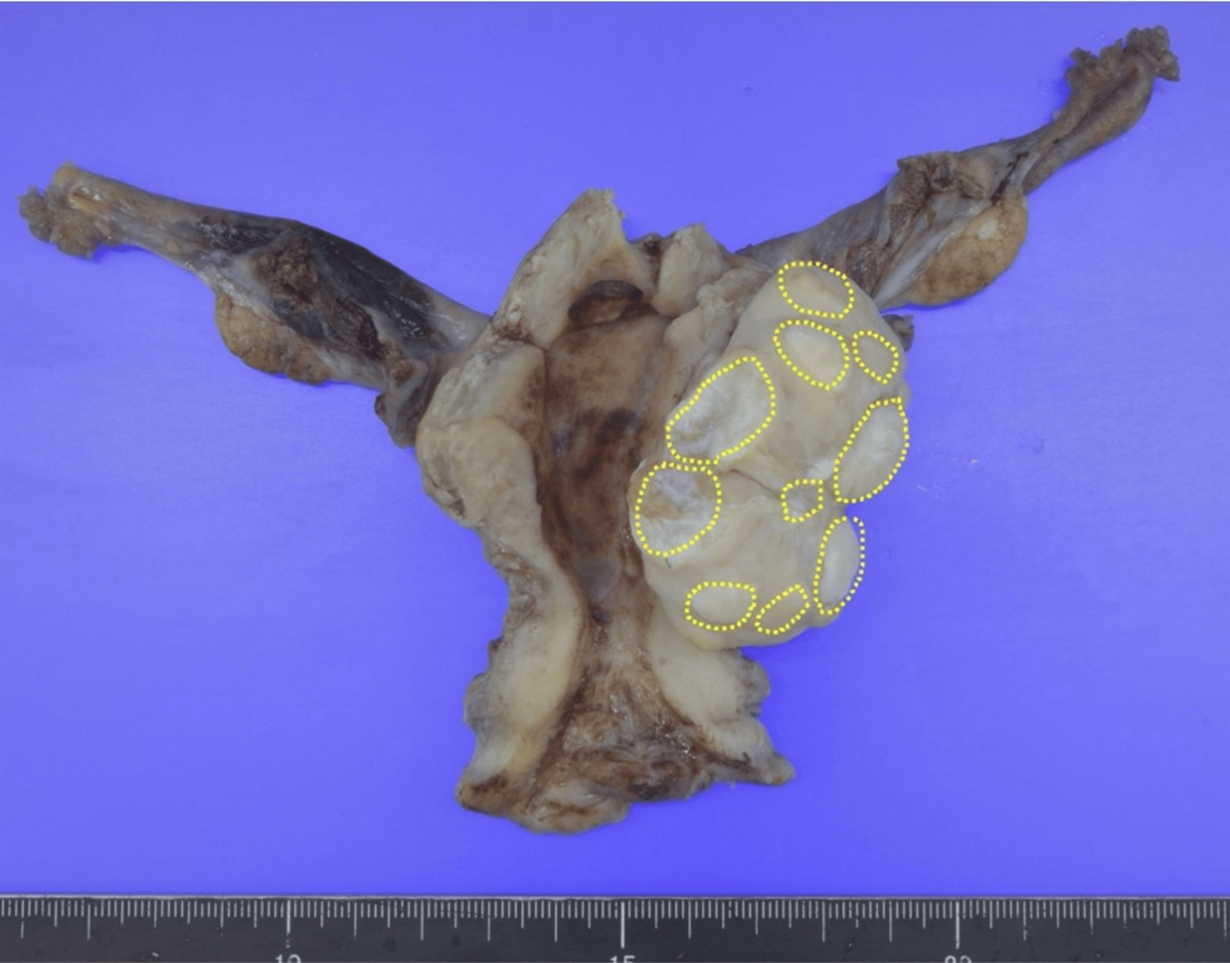
Macroscopic findings of the hysterectomy specimen A 55-mm white solid tumor with scattered yellow nodules. The distribution of low-grade endometrial stromal sarcoma is shown by the dotted line.

**Figure 5:**
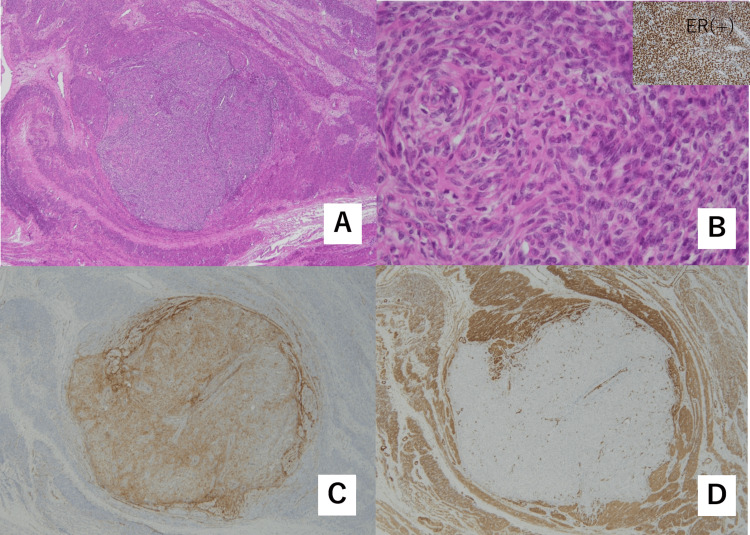
Microscopic findings of the tumor (A) A nodule is found in spindle-shaped smooth muscle cells with non-atypical nuclei (leiomyoma). (B) The nodule contains slightly atypical cells with round nuclei resembling endometrial stromal cells, which are positive for estrogen receptor (low-grade endometrial stromal sarcoma). (C and  D) Immunohistochemical staining. The nodules were positive for CD-10 (C) and negative for alpha-smooth muscle actin (D).

**Figure 6:**
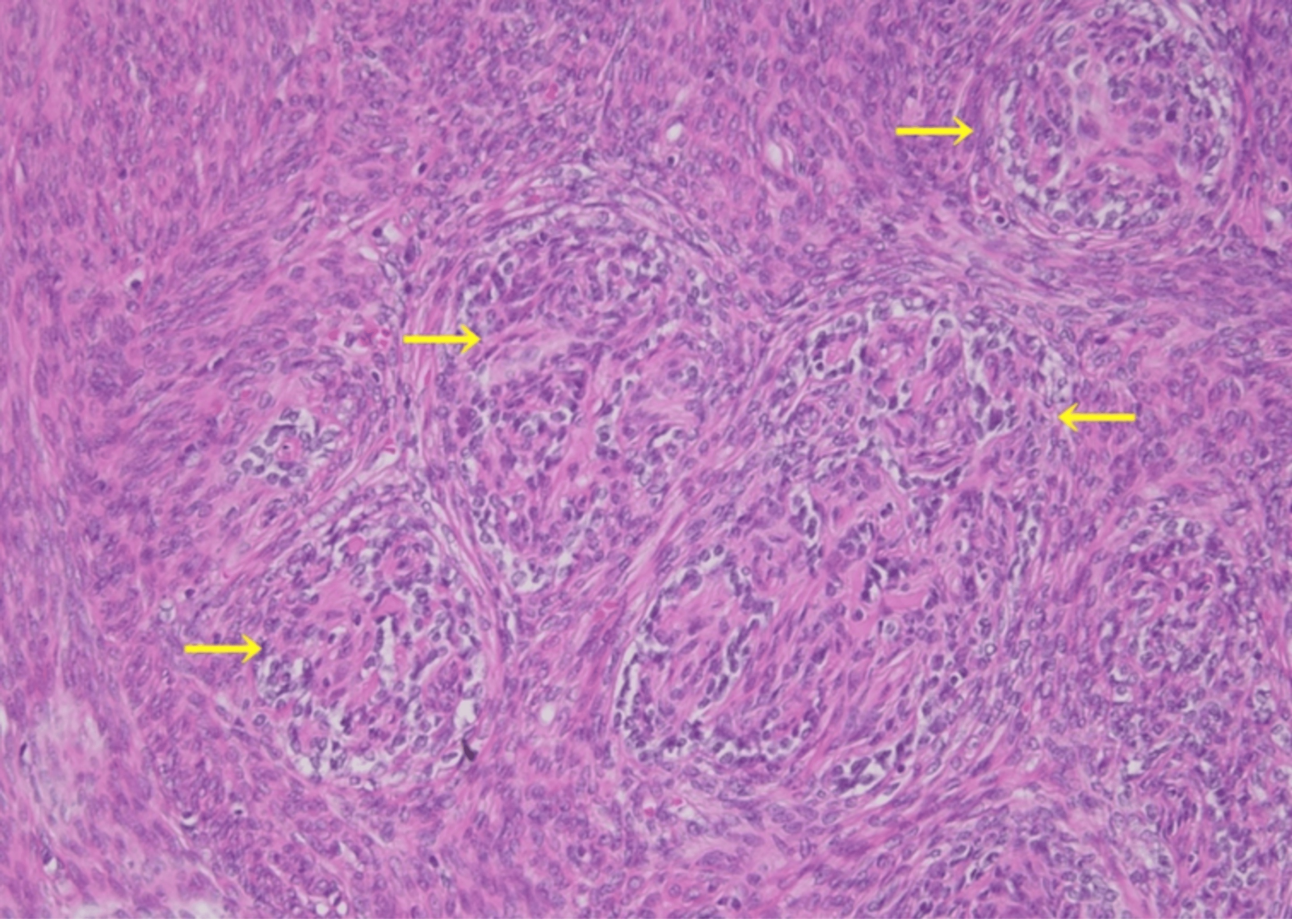
Microscopic findings of the tumor Vascular invasion of low-grade endometrial stromal sarcoma in a leiomyoma. Nests of low-grade endometrial sarcoma are shown in yellow arrows.

Macroscopic examination of the hysterectomy specimens revealed a 55-mm white solid tumor with scattered yellow nodules (Figure 4). Histopathological analysis identified spindle-shaped smooth muscle cells with non-atypical nuclei, confirming a leiomyoma. However, the tumor’s nodules contained cells with round nuclei and small nucleoli, resembling endometrial stromal cells interspersed with small blood vessels. Immunohistochemical staining showed the nodules were negative for alpha-SMA (smooth muscle actin) (0%) and positive for CD10 (Figure 5). The immunohistochemical assessment showed that Ki-67 was <1%, estrogen receptor (ER)-alpha was 100%, and progesterone receptor (PR) was 100%. These nodules invaded the leiomyoma along vascular vessels (Figure 6). The final diagnosis was leiomyoma coexisting with LGESS, classified as International Federation of Gynecology and Obstetrics (FIGO) stage IB. The patient received no further treatment. She is been followed up every six months by transvaginal ultrasonography and remains disease-free for 45 months after the surgery.

## Discussion

This is a rare case of a uterine leiomyoma lesion invaded by LGESS, the preoperative diagnosis of which was difficult. Pathological and imaging diagnoses are discussed below.

Endometrial stromal sarcoma (ESS) typically invades the myometrium and permeates it [2]. Based on our literature review, there are no prior reports of LGESS associated with leiomyoma, making this the first documented case of LGESS invading a leiomyoma. There are two types of uterine sarcomas: leiomyosarcomas arising from smooth muscle cells and ESS arising from endometrial stromal cells [2]. Both types of sarcomas develop de novo and can occur in the vicinity of existing uterine leiomyomas. It has been believed that leiomyomas do not transform into leiomyosarcomas. However, uterine leiomyosarcoma and leiomyoma have been reported to occur in the same tumor, suggesting the malignant transformation of leiomyoma into leiomyosarcoma. Evidence for this includes the histopathological continuity from leiomyomas to leiomyosarcomas [8] and genetic changes common to leiomyomas and leiomyosarcomas and new genetic changes in leiomyosarcomas [9]. However, there have been no reports on the malignant transformation from leiomyomas to ESS. The present case was diagnosed not as a malignant transformation but as ESS arising in close vicinity of an existing leiomyoma and invading it, not the myometrium, as usually seen in LGESS.

Preoperative differentiation of LGESS from leiomyomas is challenging because of the unreliability of imaging studies. In a large retrospective study, Cao et al. reported that the incidence rate of unexpected uterine sarcomas after hysterectomy and myomectomy for presumed leiomyoma was 0.33% and that the prevalence of ESS was 0.18% [6]. Leiomyoma and LGESS are both mesenchymal tumors of the uterus, with the former being a smooth muscle tumor and the latter being a endometrial stromal tumor. Leiomyoma is the most common uterine neoplasm and noted clinically in 20-30% of women over 30 years of age. LGESS is a rare tumor, accounting for only 0.2% of all malignant uterine tumors [1]. Most importantly, leiomyoma is benign, but, on the other hand, LGESS is malignant and has the potential to metastasize and recur; therefore, it requires surgery and follow-up as a malignant tumor and preoperative differentiation is important.

Typical MRI scans of LGESS show low signal intensity on T1-weighted images, high signal intensity on T2-weighted images, and high signal intensity on diffusion-weighted images [10]. LGESS invades normal muscle layers, and residual smooth muscle fibers within the tumor appear as low signal areas on T2-weighted images. Preoperative differential diagnosis of leiomyoma and LGESS is even more difficult especially when they present with an intramuscular cystic mass, as in this case report. Degeneration of leiomyoma is common, and cystic degeneration occurs in around 4% of leiomyomas [2]. Cystic lesions have also been reported in LGESS [4,5]. Therefore, differential diagnoses of myometrial cysts should include cystic degeneration of a leiomyoma [3], LGESS [5], cystic adenomyosis [11], and post-cesarean cysts, if applicable. In this case, although some MRI findings aligned with typical LGESS characteristics, the diagnosis was complicated because these features were observed within a leiomyoma, which is atypical.

Immunohistochemical staining is essential to differentiate LGESS and leiomyoma. Leiomyoma is a smooth muscle tumor and shows strong positivity for antibodies to SMA, for which LGESS may be negative or weakly positive. On the other hand, LGESS is a stromal tumor and shows strong positivity for CD10, for which leiomyoma may be negative or weakly positive. In this case, lesions of LGESS were strongly positive for CD10 and negative for alpha-SMA and were surrounded by spindle-shaped tumor cells, suggesting a leiomyoma, which were positive for alpha-SMA and negative for CD10.

We have previously reported a comparison of FDG uptake and tumor biology in recurrent and metastatic LGESS cases, four positive (maximum standardized uptake value [SUVmax] ≥ 3.0) cases in nine [7]; however, there have been limited reports on PET imaging of primary ESS cases, and Umesaki reported that one ESS (grade not reported) out of one was positive (SUV = 5.5) [12]. In the present case, SUVmax was not calculated because FDG uptake was insignificant, and the immunohistochemical assessment showed that Ki-67 was <1%, ER was 100%, and PR was 100%. This is the first report of FDG uptake and tumor biology in a primary LGESS case, as opposed to recurrent and metastatic ones. The negative FDG uptake, low Ki-67 index, and ER/PR positivity might suggest a favorable prognosis, and the patient has shown no evidence of disease for nearly four years.

Additional factors to consider in this case include the decision to perform bilateral salpingo-oophorectomy and to avoid morcellation during the hysterectomy as well as tumor enlargement following post-menopausal shrinkage. The patient was postmenopausal; therefore, bilateral salpingo-oophorectomy along with hysterectomy was performed. The uterus was small enough to be retrieved from the vagina without morcellation. We have previously reported a case of recurrent and metastatic (low-grade) ESS after laparoscopic morcellation, demonstrating the efficacy of oophorectomy for remission [13]. Other reports also suggested dissemination after morcellation for unexpected ESS [14]. With regard to bilateral oophorectomy, there have been reports of lower recurrence rates when performed simultaneously with hysterectomy [15], while other reports suggest that it has no effect on the overall survival rates [16,17].

The only clue to suspect malignancy in this case was the enlargement of the existing leiomyoma after menopause; however, the preoperative diagnosis was that the enlargement was due to cystic degeneration of the leiomyoma. The lesson in this case is that LGESS may invade the leiomyoma, and the cystic changes characteristic of LGESS may have been a clue. Cystic changes in degenerative myomas show no contrast effect on MRI and no FDG accumulation on PET. In the present case, the slight contrast effect and slight, although not significant, FDG accumulation in the small solid part of the cystic component in the leiomyoma might have been suggestive of LGESS.

## Conclusions

This is the first report of LGESS invading a leiomyoma. A cystic lesion in a leiomyoma may suggest cystic degeneration; however, it rarely indicates an LGESS invading a leiomyoma. Since LGESS is a malignant tumor and should be treated and managed differently than leiomyoma, it is important to consider the rare possibility of LGESS developing in a leiomyoma, especially if the leiomyoma enlarges after menopause. We also reported the results of a PET scan and the pathological features that may reflect tumor biology in a case of primary LGESS: slight but non-significant FDG uptake, ER/PR positivity, and low ki-67 positivity.
